# Ventricular tachycardia risk prediction with an abbreviated duration mobile cardiac telemetry

**DOI:** 10.1016/j.hroo.2023.06.009

**Published:** 2023-06-30

**Authors:** Johan Economou Lundeberg, Alexandra Måneheim, Anders Persson, Marek Dziubinski, Arun Sridhar, Jeffrey S. Healey, Magdalena Slusarczyk, Gunnar Engström, Linda S. Johnson

**Affiliations:** ∗ Department of Clinical Physiology, Skåne University Hospital, Lund, Sweden; † Department of Clinical Sciences, Lund University, Malmö, Sweden; ‡ MEDICALgorithmics, Warsaw, Poland; § University of Washington Medical Center, Seattle, Washington; || Population Health Research Institute, McMaster University, Hamilton, Ontario, Canada

**Keywords:** Ventricular tachycardia, Ambulatory ECG, Prediction, Cardiac arrythmia, Mobile cardiac telemetry, Epidemiology

## Abstract

**Background:**

Ventricular tachycardia (VT) occurs intermittently, unpredictably, and has potentially lethal consequences.

**Objective:**

Our aim was to derive a risk prediction model for VT episodes ≥10 beats detected on 30-day mobile cardiac telemetry based on the first 24 hours of the recording.

**Methods:**

We included patients who were monitored for 2 to 30 days in the United States using full-disclosure mobile cardiac telemetry, without any VT episode ≥10 beats on the first full recording day. An elastic net prediction model was derived for the outcome of VT ≥10 beats on monitoring days 2 to 30. Potential predictors included age, sex, and electrocardiographic data from the first 24 hours: heart rate; premature atrial and ventricular complexes occurring as singlets, couplets, triplets, and runs; and the fastest rate for each event. The population was randomly split into training (70%) and testing (30%) samples.

**Results:**

In a population of 19,781 patients (mean age 65.3 ± 17.1 years, 43.5% men), with a median recording time of 18.6 ± 9.6 days, 1510 patients had at least 1 VT ≥10 beats. The prediction model had good discrimination in the testing sample (area under the receiver-operating characteristic curve 0.7584, 95% confidence interval 0.7340–0.7829). A model excluding age and sex had an equally good discrimination (area under the receiver-operating characteristic curve 0.7579, 95% confidence interval 0.7332–0.7825). In the top quintile of the score, more than 1 in 5 patients had a VT ≥10 beats, while the bottom quintile had a 98.2% negative predictive value.

**Conclusion:**

Our model can predict risk of VT ≥10 beats in the near term using variables derived from 24-hour electrocardiography, and could be used to triage patients to extended monitoring.

## Graphical abstract

**Figure:**
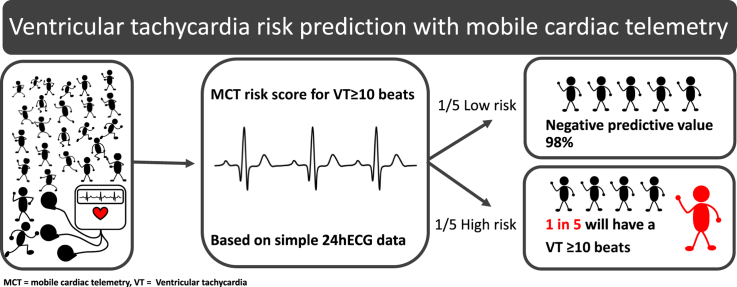

**Key Findings**

▪ A risk score based on variables from 24 hours of ambulatory electrocardiography (ECG) can predict a high risk of ventricular tachycardia (VT) ≥10 beats within 30 days and can be included in the ambulatory ECG report.
▪ In the top quintile of the risk score, a VT event with a duration of ≥10 beats was detected in 1 in 5 patients.
▪ In 20% of ambulatory ECG recordings, VT events ≥10 beats can be ruled out with a negative predictive value of 98%.

## Introduction

Ventricular tachycardia (VT) occurs intermittently, unpredictably, and can be found in both people with severe cardiovascular disease as well as apparently healthy individuals.^1^ Nonsustained VT (NSVT) episodes, defined as those lasting <30 seconds, have been associated with poor prognosis and sudden cardiac death in several different patient cohorts.^2^, ^3^, ^4^ NSVT episodes are associated with a doubled risk of sudden cardiac death after myocardial infarction,^3^ and it has been argued that patients with NSVT after an acute coronary syndrome should receive a more thorough evaluation.^5^ The presence of NSVT is also integral for evaluation of symptoms, as well as for evaluation of treatment response after initiation of antiarrhythmic drugs, or post–VT ablation. NSVT episodes are additionally a component of the recently published hypertrophic cardiomyopathy risk prediction model suggested by the European Society of Cardiology, as well as the Montreal Arrhythmogenic Right Ventricular Cardiomyopathy risk prediction rule.^6^^,^^7^

Finally, subjects with incidentally detected NSVT episodes have a doubled risk of mortality and cardiovascular events in a population without known structural heart disease.^8^ Early detection could potentially lead to improved risk factor control to prevent adverse outcomes.

Mobile cardiac telemetry (MCT) recordings are indicated as part of the diagnostic workup for a diverse range of symptoms, including chest palpitations, syncope, and near syncope.^9^ The optimal monitoring duration for these indications remains unknown; while short recording durations imply a risk of underdetection of significant arrhythmias,^10^ longer recordings are associated with increased cost and patient discomfort as well as incidental detection of arrythmias without clinical relevance.^10^^,^^11^ Currently, data collected during short MCT recordings are not used to inform the need for extended or repeated monitoring, but a computer-derived risk score could be used by physicians to triage patients to shorter or longer monitoring durations depending on the clinical situation.

## Methods

We included all patients who were monitored in the United States using the PocketECG device (MEDICALgorithmics, Warsaw, Poland) for 2 to 30 full days (N = 19,947) in 2020. We excluded 158 patients with a VT event ≥10 beats on the index day, defined as the first full day of recording, as well as 8 patients >100 years of age, resulting in a final study population of 19,781 patients monitored for a clinical indication. All arrhythmias were algorithmically detected and manually verified by trained electrocardiography (ECG) technicians. The Swedish Ethical Review Authority has waived the need for ethics approval for studies using this database because all analyses are performed on retrospective and fully anonymized data. The study conforms to the Declaration of Helsinki.

Potential predictors were derived from the ECG collected during the index day and included measures of heart rate during sinus rhythm (minimum and maximum heart rates measured as 1-minute averages and mean heart rate overall), as well as the prevalence and pattern of occurrence of premature atrial complexes (PACs) and premature ventricular complexes (PVCs). PACs and PVCs were included as the total number of each beat type as well as their occurrence as singlets, couplets, and triplets. Supraventricular tachycardia (SVT) and VT were defined as 4 or more consecutive PACs or PVCs, respectively, with a frequency ≥100 beats/min. Accelerated idioventricular rhythm was defined as an episode of 4 or more broad QRS-complexes without detectable P waves and a frequency <100 beats/min. Bradycardia was defined as sinus rate <50 beats/min with a minimum duration of 1 minute. We also included the fastest rate of each arrhythmic event class, derived from the shortest pair-interval rate, for all arrhythmias lasting ≥2 beats. To avoid the introduction of missing data to the model for variables describing heart rate during arrhythmia, the fastest heart rate was set to 0 for those without any occurrences of the arrhythmia in question. Mean, maximum, and minimum heart rate were calculated based on sinus beats only, excluding the time spent in any arrhythmia, and measured over 1-minute durations in beats per minute. The outcome was defined as a VT event with a duration ≥10 beats (VT ≥10 beats), occurring any day after the index day (registration days 2–30).

### Statistical methods

All statistics were performed using Stata17.0 (StataCorp, College Station, TX). The study population was randomly split into training (70%) and testing (30%) datasets, and a prediction model for VT ≥10 beats was derived using elastic net, with selection of the best model by 10-fold cross-validation. We assessed several potential prediction models. In a full model, we specified 28 potential predictors, including age, sex, and ECG data from the first 24 hours: heart rate (minimum, maximum, and mean); lowest rate during bradycardia; the number of PACs and PVCs, occurring as singles, couplets, and triplets; the fastest rate during each of these arrhythmias; and maximal heart rate as well as the duration of SVT, VT, and accelerated idioventricular rhythm episodes. We also derived a second ECG-only model without age and sex. These models were compared with an age- and sex-only model, as well as a fourth model that included only the total PVC count on the index day.

β coefficients for the main model and the ECG-only model were used to calculate individual predicted risks of VT ≥10 beats, and the resulting predicted risk scores were included as a single predictor in a logistic regression model in the testing sample. Model discrimination was assessed using receiver operating characteristic (ROC) curves and area under the ROC curve with 95% confidence intervals (CIs). Model calibration was assessed with the Hosmer-Lemeshow test using 10 equal-sized bins. The results were plotted visually with estimation of calibration-in-the-large, slope, and expected-to-observed ratio using the Stata plugin pmcalplotevents, downloaded from Boston College Statistical Software Components (https://www.stata.com/support/ssc-installation/). In order to estimate the occurrence of sustained VT episodes, we identified the longest VT for each patient and estimated sustained VT as any VT with a duration ≥60 beats, which we consider to be a conservative estimate.

## Results

The mean age was 65.3 ± 17.1 years (43.5% male), with an age range of 17 to 100 years. The mean monitoring duration was 18.6 ± 9.6 days. A total of 27%, 27%, and 46% had a recording time of between 1 and 10 days, 11 and 20 days, and 21 and 30 days, respectively. VT ≥10 beats occurred in 1510 (7.6%) patients, after a median recording time of 10 (interquartile range 4–19) days. In patients with VT ≥10 beats, the median duration of the longest VT was 13 (interquartile range 10–258) beats. A total of 93 patients had at least 1 VT episode ≥25 beats. Twenty patients had at least 1 sustained VT. VT ≥10 beats were more common among men compared with women (10.3% vs. 5.6%). Population statistics are reported in more detail in Table 1.

**Table 1:** Study population characteristics

Age, y	65.3 ± 17.1
Male, %	43.5
Heart rate, beats/min	73.5 ± 12.0
Maximum heart rate, beats/min	113.7 ± 22.1
Minimum heart rate, beats/min	56.3 ± 9.8
Total beats	95,333 ± 21,641
Total PACs	70 (12–659)
Single PACs	37 (7–229)
PAC couplets	1 (0–5)
PAC triplets	0 (0–2)
SVTs ≥4 beats	0 (0–2)
AIVR beats	0 (0–0)
Total PVC count	22 (2–261)
Single PVC count	21 (2–254)
PVC couplets	0 (0–1)
PVC triplets	0 (0–0)
VT runs ≥4 beats	0 (0–0)

Values are mean ± SD or median (interquartile range), unless otherwise indicated.

AVIR = accelerated idioventricular rhythm; PAC= premature atrial complex; PVC = premature ventricular complex; SVT = supraventricular tachycardia; VT = ventricular tachycardia.

### Prediction model discrimination, calibration, and goodness of fit

Of 28 potential predictors, 14 variables were selected in the full model and 16 variables were selected in the ECG-only model. The selected variables for both models are available in the Supplemental Appendix (Table 1), along with the corresponding β coefficients and the formula used for calculation of predicted risk.

**Figure 1:**
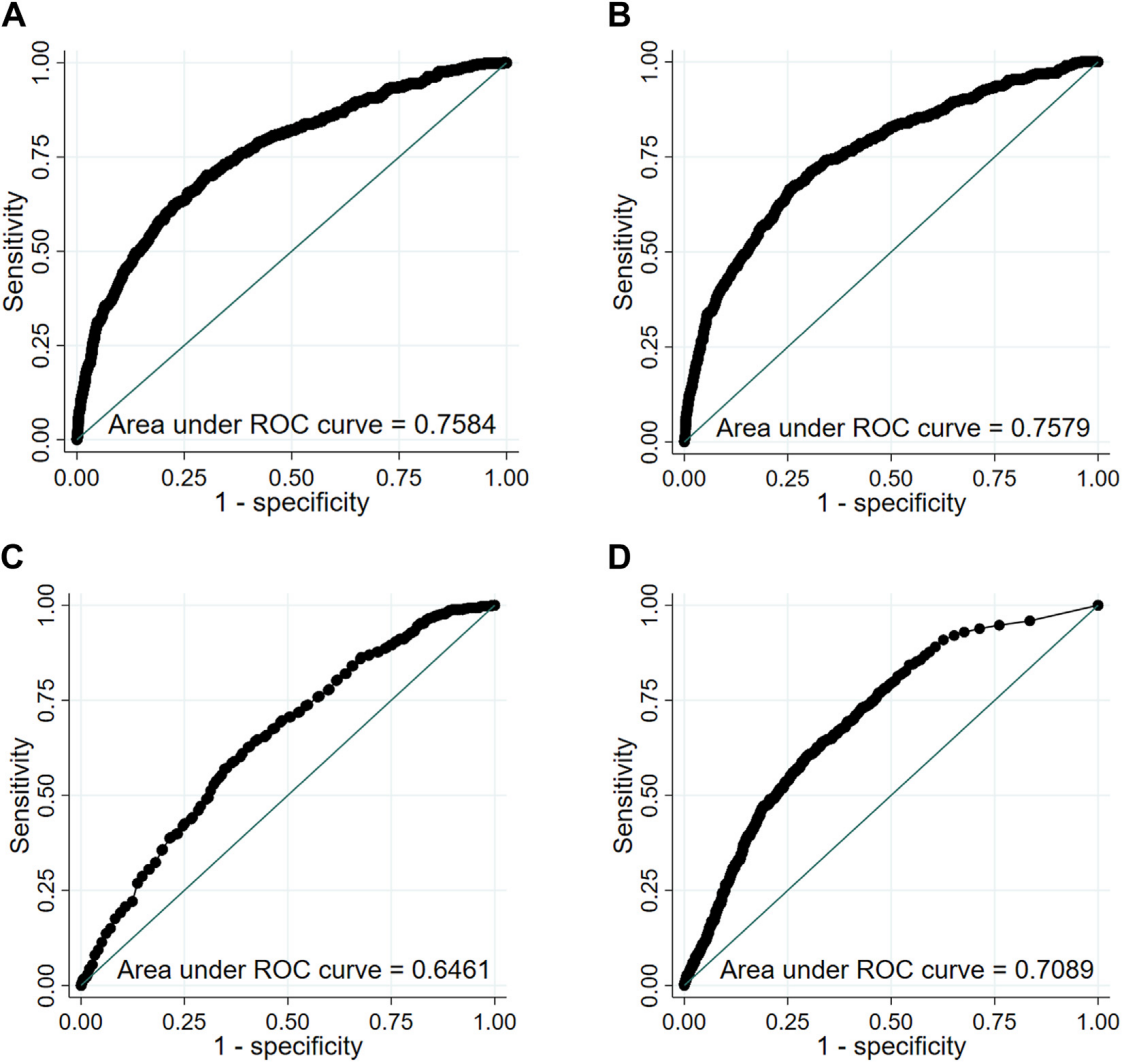
Receiver-operating characteristic (ROC) curves for ventricular tachycardia ≥10 beats occurrence by predicted risk in the testing sample. **A:** Main model; **B**: electrocardiography-only model; **C:** age and sex model; **D:** premature ventricular complex model.

Both the full model and ECG-only model had good discrimination in the testing sample: areas under the ROC curve of 0.7584 (95% CI 0.7340–0.7829) and 0.7579 (95% CI 0.7332–0.7825), respectively (Figure 1A and 1B). Observed events by predicted risk quintiles in the testing data set are reported for both the main and ECG-only models in Table 2. For both models, the observed risk was within the range of predicted risk in all quintiles except the third (in which risk was somewhat lower). In the top quintile for predicted risk, sex was more evenly distributed for the ECG-only model compared with the full model (male sex 56.4% vs. 69.7%). In the bottom quintile, the negative predictive value for the full model was 98.0% and for the ECG-only model was 98.2%. All sustained VT events were detected in the top quintile of predicted risk both models.

**Table 2:** Observed and predicted VT events ≥10 beats by quintiles of predicted risked in the testing dataset (n = 5934)

Variable	Q1 (n = 1186)	Q2 (n = 1187)	Q3 (n = 1187)	Q4 (n = 1187)	Q5 (n = 1187)
Main model					
Predicted risk, %	1.5–3.3	3.3–4.6	4.6–6.1	6.1–10.1	>10.1
At least 1 VT ≥10 beats	24 (2.0)	40 (3.4)	46 (3.9)	90 (7.6)	239 (20.1)
Age, y	41.3 ± 14.9	64.3 ± 13.3	71.0 ± 9.6	74.2 ± 10.6	75.3 ± 8.9
Male	241 (20.3)	331 (27.9)	497 (41.9)	694 (58.5)	827 (69.7)
NPV, %	98.0	96.6	96.1	92.4	79.9
ECG-only model					
Predicted risk, %	1.8–3.5	3.5–4.5	4.5–6.0	6.0–10.0	>10.0
At least 1 VT ≥10 beats	21 (1.8)	43 (3.6)	46 (3.9)	93 (7.8)	236 (19.9)
Age, y	45.1 ± 17.5	64.1 ± 15.0	70.5 ± 11.8	72.2 ± 11.9	74.3 ± 9.6
Male	457 (38.5)	506 (42.6)	450 (37.9)	508 (42.8)	669 (56.4)
NPV, %	98.2	96.5	95.8	92.7	80.2

Values are n (%) or mean ± SD, unless otherwise indicated.

NPV = negative predictive value; Q = quartile; VT = ventricular tachycardia.

The output of the model is a predicted risk that assumes continuous values between 0% and 100%, and we consider this to be the most useful way to consider the data. However, we also calculated the maximally predictive point, based on the Youden method. With this method, the optimal cutoff for the main model was at a sensitivity of 70% and a specificity of 70% in the testing sample. For the ECG-only model, the optimal cutoff yielded a sensitivity of 67% and a specificity of 74%.

A model based only on age and sex, or only total PVC count had substantially worse discrimination: areas under the ROC curve of 0.6461 (95% CI 0.6208–0.6715) and 0.7089 (95% CI 0.6855–0.7324), respectively (Figure 1C and 1D).

There was no sign of over- or underfitting of the data, for neither the full model nor the ECG-only model (slope = 1.070, intercept = –0.068 for the full model; slope = 1.089, intercept = –0.066 for the ECG-only model). Calibration plots for all 4 models are available in the Supplemental Appendix (Figure 1).

Subsequently, we examined patients with a minimum of 14 days of ambulatory ECG to assess the model's performance. In the testing sample, the main model demonstrated an area under the ROC curve of 0.7487 (95% CI 0.7216–0.7759) with acceptable discrimination (slope = 1.082, intercept = 0.214).

## Discussion

Using only data easily derived from 24 hours of MCT, we derived a risk prediction model for NSVT episodes ≥10 beats on MCT monitoring in the next 29 days. In the top quintile of the predicted risk score roughly 1 in 5 patients had an episode of VT ≥10 beats during the subsequent monitoring. In the bottom quintile, the negative predictive value was 98% for both models.

This model could be useful both to identify low- and high-risk patients. In instances of abbreviated duration, the risk score can assist in determining if a renewal of the MCT is necessary. For high-risk patients, extended or renewed monitoring might be indicated depending on the clinical situation. The prediction algorithm could also be useful as an integrated automatic tool to alert physicians to high VT risk when patients are monitored with shorter MCT recordings for other indications. In patients in whom an incidental finding of NSVT would be clinically relevant, the monitoring duration could then be extended. In areas with limited resources and device availability, prediction models such as these can be used to identify which patients are more likely to benefit from prolonged monitoring.

MCT has been commercially available since 1963 for detection and quantification of arrythmias such as VT.^12^ The clinical relevance and interpretation of VTs found on MCT are under scientific discussion. In specific patient groups, such as patients with hypertrophic cardiomyopathy, left ventricular dysfunction, and after hospitalization due to acute coronary syndrome, VT has been linked to cardiac mortality.^2^, ^3^, ^4^^,^^13^ Conflicting results have nevertheless been found in which nonsymptomatic VT detected by pacemaker interrogation has not been found to be independently associated with mortality.^14^ However, in the 2019 European Heart Rhythm Association/Heart Failure Association/Heart Rhythm Society consensus document for the management of asymptomatic arrhythmias, it is recommended that all patients with asymptomatic NSVT should undergo an evaluation to detect underlying heart disease.^15^ Consistent with these guidelines, a recently published study from the population-based Copenhagen Holter study showed that incidentally detected nonsustained VT was associated with a doubled risk of mortality and cardiovascular events.^8^ We therefore believe that this MCT-based VT risk score could have clinical utility.

In recent years, there has been an increasing research interest in the clinical significance of relatively low levels of PVCs, as they have been shown to predict future risk of heart failure and mortality.^16^^,^^17^ However, PVCs, and by extension VTs, are a heterogeneous group of arrythmias with varying underlying mechanisms, and one could therefore argue that it is not enough to classify PVCs and VTs on quantity alone.^18^^,^^19^ Other characteristics such as QRS duration, mono- or multiform complexes, morphology, and coupling interval to previous normal beat have been suggested as novel predictors of cardiovascular disease.^20^, ^21^, ^22^ To create a simple and parsimonious model, these variables have not been included in the present risk score, but it is possible that such detailed ECG-derived data could be implemented in future models and that this could prove valuable for prediction not only of VT, but also of incident cardiac events and mortality.

Risk assessment based on an MCT examination result is often not straightforward for physicians in different clinical settings. Predictive analytics for specific arrhythmias or diseases incorporated in an MCT report could help physicians make more informed decisions about whether to pursue extended monitoring or other interventions. However, a computer-derived risk score should always be used as a tool to aid clinical decision making, rather than as a substitute for physician judgment. Ultimately, the decision to pursue extended monitoring should always be made on a case-by-case basis.

### Limitations

This study has some limitations that should be kept in mind when interpreting the results. Considering the lack of sufficient scientific evidence of what defines a clinically relevant VT, the chosen cutoff at ≥10 beats was based on clinical expertise as well as the 2020 American Heart Association/American College of Cardiology guidelines for management of hypertrophic cardiomyopathy.^23^

Because the data for this study were shared by the device manufacturer, no clinical data were available, beyond age and sex, and this has limited our ability to test the derived model in specific patient subgroups that would be of relevance, for example patients with and without coronary artery disease or heart failure. We have also been unable to assess whether the score predicts sustained VT episodes or clinical outcomes, including death from cardiovascular causes. However, by using a large, unselected patient material from the United States, we believe that our model is based on a population that is generalizable to diverse patient cohorts. Future studies assessing the performance and clinical utility of the score in defined patient populations would be of value.

## Conclusion

A risk score based on variables from 24 hours of MCT can predict a high risk of VT ≥10 beats within 30 days and can be used to triage patients according to their need for extended monitoring. In the top quintile of the risk score, a VT event with a duration of ≥10 beats was detected in 1 in 5 patients.

## Appendix Supplementary Data

Supplemental_Data

Risk prediction calculator
