# Supplementary material for: Ventricular tachycardia risk prediction with an abbreviated duration mobile cardiac telemetry
Source: Heart Rhythm O2. 2023 Jun 30;4(8):500–5. doi: 10.1016/j.hroo.2023.06.009 (PMC10461200; doi:10.1016/j.hroo.2023.06.009)
Supplement: Supplemental_Data [file mmc1.docx]

**Appendix**

**Table A.1** Derived ß-coefficients for risk prediction models of VT≥10 beats.

|  | Main model | ECG-only model |
| --- | --- | --- |
|  | ß-coefficient | ß-coefficient |
| Age, per year | .0128373 |  |
| Sex (Female=1, Male=2) | .4037586 |  |
| Mean heart rate, per bpm |  |  |
| Maximum heart rate, per bpm | -.0016646 | -.006056 |
| Minimum heart rate, per bpm |  |  |
| Lowest rate during bradycardia, per bpm | -.0018789 | -.0028304 |
| Total number of beats, per 10’000 beats |  | -.0026697 |
| Total PAC count, per 1000 beats |  | .0017096 |
| Single PAC count, per 1000 beats |  |  |
| Number of PAC couplets, per 10 couplets |  |  |
| Fastest rate during PAC couplet, per bpm | .0013599 | .002136 |
| Number of PAC triplets, per 100 triplets |  | -.0022087 |
| Fastest rate during PAC triplet, per bpm |  |  |
| Number of SVTs≥4 beats |  |  |
| Longest duration of SVT, per beat |  |  |
| Fastest rate during SVT≥4 beats, per bpm | .0019014 | .0020498 |
| Total number of AIVR events |  |  |
| Fastest rate during AIVR, per bpm | .002189 | .0026436 |
| Longest duration of AIVR, per beat |  | -.0000738 |
| Total PVC count, per 1000 beats | -.003664 | -.0021928 |
| Single PVC count, per 1000 beats |  |  |
| Total number of PVC couplets, per 100 couplets | -.0039989 | -.0021845 |
| Fastest rate during PVC couplet, per bpm | .0052199 | .006003 |
| Number of PVC triplets, per 10 triplets | -.0011753 | -.004129 |
| Fastest rate during PVC triplet, per bpm | .0058987 | .0062653 |
| VT runs≥4 beats | .0023373 | .0031469 |
| Fastest rate during VT run≥4 beats, per bpm |  |  |
| Longest duration of VT run, per beat | .1660528 | .1657541 |
| ß_0_ (Model intercept) | -4.42906 | -2.540892 |

Predicted risk for the models can be calculated as *P=e^(β^_0_^+β^_1_^X^_1_^+β^_2_^X^_2_^+….+β^_n_^X^_n_ /(1+e^(β0+β^_1_^X^_1_^+β^_2_^X^_2_^+….+β^_n_^X^_n_^)^* )

AVIR = accelerated idioventricular rhythm, Bpm= beats per minute, PAC= premature atrial complex, PVC= premature ventricular complex, SVT= supraventricular tachycardia, VT= ventricular tachycardia.

**Figure A.1** Calibration plots for VT≥10 prediction in the testing sample.

BECG only model

AECG only model

CECG only model

DECG only model

A = Main model, B = ECG-only model, C = Age and sex model, D = Premature ventricular complex model

E:O = Expected/observed ratio, CITL = Calibration-in-the-large, AUC = Area under the curve
